# Reperfusion‐Dependent Outcomes After Endovascular Thrombectomy Stratified by NIHSS‐ASPECTS Clinical‐Core Mismatch

**DOI:** 10.1002/acn3.70358

**Published:** 2026-03-12

**Authors:** Felix Schlicht, Lukas Meyer, Gabriel Broocks, Matthias Bechstein, Christian Thaler, Christian Heitkamp, Laurens Winkelmeier, Vincent Geest, Alexander Heitkamp, Maximilian Jungnitz, Gregor Peter, Luca Meucci, Tobias Faizy, Jawed Nawabi, Caspar Brekenfeld, Fabian Flottmann, Maximilian Schell, Alexandros Hadjilaou, Uta Hanning, Götz Thomalla, Jens Fiehler, Susanne Gellißen, Helge C. Kniep, German Stroke Registry Endovascular Treatment, (GSR‐ET) Alexander Nave, Anna Alegiani, Annerose Mengel, Arno Reich, Bernd Eckert, Charlotte Pietrock, Christian Nolte, Eberhard Siebert, Fabian Flottmann, Fee Keil, Franziska Dorn, Gabor Petzold, Götz Thomalla, Hanna Zimmermann, Jan Borggrefe, Jan Hendrik Schäfer, Jens Fiehler, Joachim Röther, Klaus Gröschel, Lars Kellert, Mario Abruscato, Maximilian Schell, Omid Nikoubashman, Peter Schellinger, Silke Wunderlich, Steffen Tiedt, Sven Thonke, Timo Uphaus, Tobias Boeckh‐Behrens, Ulrike Ernemann

**Affiliations:** ^1^ Department of Diagnostic and Interventional Neuroradiology University Medical Center Hamburg‐Eppendorf Hamburg Germany; ^2^ Department of Neuroradiology HELIOS Medical Center, Campus of MSH Medical School Hamburg Schwerin Germany; ^3^ Neuroendovascular Program, Department of Radiology University Hospital Muenster Muenster Germany; ^4^ Department of Neuroradiology Charité Medical Center Berlin Germany; ^5^ Department of Neurology University Medical Center Hamburg‐Eppendorf Hamburg Germany

**Keywords:** clinical‐Core mismatch, national institutes of health stroke scale, reperfusion, stroke, thrombectomy

## Abstract

**Objective:**

This analysis evaluates the effect of successful reperfusion on functional outcomes after MT, stratified by admission National Institutes of Health Stroke Scale (NIHSS) and Alberta Stroke Program Early CT Score (ASPECTS) as surrogates for clinical‐core mismatch, using multicenter registry data.

**Methods:**

Retrospective analysis of patients with anterior circulation stroke undergoing MT enrolled in the German Stroke Registry (2015–2023). Patients were stratified into nine subgroups according to ASPECTS (10; 9–8; ≤ 7) and NIHSS (0–10; 11–15; ≥ 16). The primary endpoint was the rate of good functional outcome (modified Rankin Scale [mRS] of 0–2) at 90 days. Safety outcomes included symptomatic intracranial hemorrhage and mortality. Inverse‐probability‐weighted regression adjustment (IPWRA) was used to control confounders in observational data.

**Results:**

18,069 patients were screened, 5448 met the inclusion criteria (mean age 71.2; 48% female). Successful reperfusion (mTICI 2b‐3) was associated with improved functional outcomes in all subgroups, including patients with low NIHSS and high ASPECTS or high NIHSS and low ASPECTS. Largest effects were observed for high clinical‐core mismatch (ASPECTS = 10; NIHSS ≥ 16) with 47% (95% Confidence Interval [CI]: 42%–53%) mRS 0–2 after mTICI 3 recanalization compared to 9% (95% CI: 3%–16%) after persistent occlusion, a 38‐percentage point increase in good outcome. mTICI 3 was associated with superior outcomes compared to mTICI 2b, particularly in patients with high NIHSS and high ASPECTS.

**Interpretation:**

Patients with pronounced clinical‐core mismatch derive the greatest benefit from successful reperfusion. Nonetheless, significant functional improvements across all subgroups support more individualized treatment considerations and highlight the need for refined selection criteria.

## 1 Introduction

Mechanical thrombectomy (MT) has been established as a fundamental treatment modality for acute ischemic stroke due to large vessel occlusion [1]. Currently, the boundaries of endovascular MT continue to expand [2]. Recent randomized controlled trials (RCTs) showed that MT improves functional outcome compared to medical treatment alone in patients with large infarcts, characterized by low Alberta Stroke Program Early CT Scores (ASPECTS) at admission [2, 3, 4, 5, 6, 7, 8]. Conversely, the potential role of MT in patients presenting with mild neurological deficits, defined by low NIHSS scores, has gained the focus of ongoing investigations (3, 4, 8). It is anticipated that further insights will emerge from currently ongoing trials such as ENDOLOW (Endovascular Therapy for Low NIHSS Ischemic Strokes) [3], and MOSTE (Minor Stroke Therapy Evaluation) [4].

Previous studies showed that effects of reperfusion after MT are influenced by the interplay between the volume of recoverable tissue (penumbra) and the irreversible infarcted regions (infarct core) within the hypoperfused vascular territory [9]. Conventionally, patients with substantial volumes of potentially recoverable brain tissue can be identified either through perfusion imaging or clinically by the presence of a disproportionate severe neurological deficit relative to the extent of demarcated infarction, referred to as clinical‐core mismatch [10, 11]. For patients with high clinical‐core mismatch, an increased responsiveness to MT and higher probability of good functional outcome has been reported [10]. However, the recently observed benefit of successful reperfusion in patients with initially large infarcts, alongside the uncertain efficacy in those with small infarcts and mild clinical symptoms, highlights ongoing questions regarding the mechanisms underlying outcome variability and the role of clinical‐core mismatch as a prognostic indicator. Despite findings from recent RCTs enrolling specific subgroups, it is still uncertain whether the positive effects of successful reperfusion can be reliably reproduced across more diverse patient populations in routine clinical settings.

This study based on large‐scale multicenter registry data systematically assesses reperfusion‐dependent effects after MT across distinct clinical profiles stratified by ASPECTS and NIHSS, including patients with both high and low clinical‐core mismatch. We hypothesize that the reperfusion‐dependent effects on outcomes following MT in this real‐world cohort reflect findings from clinical trials, with greater benefit observed in patients exhibiting a high clinical‐core mismatch.

## 2 Methods

### 2.1 Study Design

This retrospective multicenter cohort study analyzed data from patients prospectively enrolled in the GSR‐ET between June 2015 and December 2023. The GSR‐ET is an ongoing, prospective, open‐label, multicenter registry that includes patients undergoing MT at 25 comprehensive stroke centers across Germany (ClinicalTrials.gov identifier: NCT03356392). Ethical approval for the registry was granted by the Ethics Committee at Ludwig Maximilian University, Munich, Germany (689–15), as well as by local ethics committees at each participating site in accordance with their respective regulations. Written informed consent was obtained from every participant (or their legally authorized representative) in accordance with the Declaration of Helsinki. A comprehensive description of the GSR‐ET study design and the major findings has been published previously [12, 13].

The main inclusion criteria of GSR‐ET were diagnosis of acute ischemic stroke due to large vessel occlusion, initiation of an endovascular procedure for treatment, and age ≥ 18 years. There are no exclusion criteria. This research was conducted in accordance with the Strengthening the Reporting of Observational Studies in Epidemiology (STROBE) guidelines for observational studies [14]. All procedures complied with the principles of the Declaration of Helsinki [15].

### 2.2 Study Cohort

**FIGURE 1:**
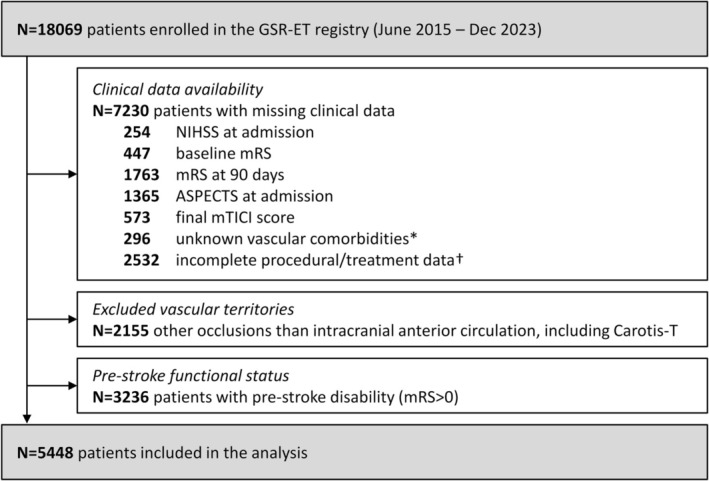
Patient Selection and Cohort Derivation Flowchart. Patients were excluded due to missing core clinical/imaging variables, occlusions outside the MCA territory, or prestroke functional dependence (mRS > 0). Further reasons included: *missing data on vascular comorbidities: Hypertension *n* = 79, diabetes *n* = 87, dyslipidemia *n* = 71, atrial fibrillation *n* = 59. †incomplete treatment‐related and procedural data: Antithrombotic treatment *n* = 1191, anesthesia *n* = 375, mRS difference > 0 *n* = 199, outside or unknown MCA territory *n* = 33, age *n* = 16, sex *n* = 12, number of passes ≤ 15 *n* = 706. Abbreviations: ASPECTS: Alberta Stroke Program Early CT Score; mRS: Modified Rankin Scale; mTICI: Modified Thrombolysis in Cerebral Infarction; NIHSS: National Institutes of Health Stroke Scale; tPA: Tissue plasminogen activator.

All patients prospectively enrolled in the GSR‐ET from June 2015 to December 2023 were screened. All patients with anterior circulation occlusions were included. Of those, patients with extracranial internal carotid artery (ICA) occlusions and with pre‐stroke disability, defined as a modified Rankin Scale (mRS) score > 0, were excluded. The complete case analysis additionally excluded patients with missing key clinical data, including the baseline National Institutes of Health Stroke Scale (NIHSS) and Alberta stroke program early CT (ASPECTS) scores, information on comorbidities, antithrombotic medication, administration of intravenous thrombolysis, anesthetic management, number of thrombectomy passes, recanalization success, and functional outcome at 90 days. A patient inclusion flowchart is provided in (Figure 1).

### 2.3 Clinical and Radiological Data Acquisition

All clinical and imaging parameters including NIHSS, ASPECTS, and mRS were reported by local investigators at each participating site [12, 13]. Baseline imaging, digital subtraction angiography, and follow‐up imaging were assessed locally. The recanalization status was determined by the treating neurointerventionalist using the modified treatment in cerebral infarction (mTICI) score [16]. ASPECTS was primarily assessed on non‐contrast CT. Diffusion‐weighted MRI was used when CT was inconclusive or when MRI was the primary admission modality. Successful reperfusion was defined as a final mTICI score of 2b–3, full recanalization as mTICI 3. A separate analysis of mTICI 2c was not performed because differentiation between 2b and 2c was inconsistently documented across registry sites during the early registry period. Clinical status was evaluated at admission, after 24 h, and after 90 days using the NIHSS and mRS.

### 2.4 Outcome Measures

The primary endpoint was good functional outcome, defined as an mRS score of 0–2 at 90 days after treatment [17, 18]. Safety outcomes included symptomatic intracranial hemorrhage (sICH), defined according to ECASS II (European Cooperative Acute Stroke Study‐II) criteria as any intracranial hemorrhage within 24 h accompanied by an increase of at least 4 points on the NIHSS [19] and mortality within 90 days after stroke. Exploratory analysis included assessment of mean mRS scores within subgroups and a linearized mRS shift analysis at 90 days post‐treatment, reflecting changes in stroke‐related disability relative to pre‐stroke functional status.

### 2.5 Statistical Analysis

Descriptive statistics were used to summarize patient characteristics and study endpoints. Continuous variables were reported as means with standard deviations (SD), ordinal variables as medians and interquartile ranges (IQR), and categorical variables as frequencies and percentages. Group comparisons were performed using one‐way analysis of variance (ANOVA) test for continuous variables, Kruskal‐Wallis test for ordinal variables, and chi‐square test for categorical variables. Reperfusion‐dependent effects on outcomes were evaluated using binarized metrics (primary endpoint, increase in the proportion of good outcome mRS 0–2 at 90 days; safety endpoints, mortality and rate of sICH) and linearized metrics (exploratory analysis, mean mRS at 90 days and mRS shift). Recanalization success was categorized into three groups: unsuccessful reperfusion (mTICI 0–2a), partial reperfusion (mTICI 2b), and complete reperfusion (mTICI 3). Subgroup analyses for evaluation of effects on outcomes across distinct clinical profiles included nine subgroups stratified by ASPECTS (10, 9–8, ≤ 7) and NIHSS (0–10, 11–15, ≥ 16). Average outcomes across treatment levels were estimated using double‐robust inverse‐probability‐weighted regression adjustment (IPWRA) to control for confounding in observational data. Confounding variables were selected based on statistical significance in univariate regression. Outcome models were controlled for age, sex, use of antithrombotic medication, comorbidities diabetes, hypertension, atrial fibrillation, and dyslipidemia, NIHSS at admission, ASPECTS at admission, administration of intravenous thrombolysis, and number of thrombectomy passes. Treatment models with the dependent variable recanalization success were controlled for age, sex, use of antithrombotic medication, comorbidities hypertension, atrial fibrillation, and dyslipidemia, NIHSS at admission, ASPECTS at admission, and number of thrombectomy passes. Time from symptom onset to imaging was not consistently available across registry entries and therefore not incorporated into the IPWRA models. All statistical analyses were performed using Stata/MP version 18.0 (Stata/MP18, StataCorp, TX, USA), with statistical significance defined as *p* < 0.05. The corresponding author had full access to all data in the study and takes responsibility for the integrity of the data and the accuracy of the data analysis.

## 3 Results

### 3.1 Baseline Characteristics

18,069 patients enrolled in the GSR between June 2015 to December 2023 were screened. A total of 5448 patients were included in the analysis, thereof 650 with final mTICI 0–2a, 1873 with final mTICI 2b, and 2925 with final mTICI 3 (Figure 1). Mean age was 71.2 years (SD 13.1) and 48% of patients were female. NIHSS at admission was comparable across groups, with a median of 13 (IQR 8–18). Admission ASPECTS differed significantly between groups (*p* < 0.001), with the highest scores observed in patients achieving complete recanalization mTICI 3 (median 9, IQR 7–10) and the lowest in those with minimal or no reperfusion mTICI 0–2a (median 8, IQR 7–10). Intravenous thrombolysis was administered in 54% of patients, more often in cases with mTICI 3 (54%) and mTICI 2b (55%) recanalization than in mTICI 0–2a (49%) (*p* = 0.028). The mean number of retrieval attempts was lowest in the mTICI 3 group (1.8, SD 1.2) and highest in the mTICI 0–2a group (3.0, SD 2.5), group comparison *p* < 0.001. General anesthesia was more frequently used in patients with successful reperfusion, with the highest number in the mTICI 3 group (77%), followed by mTICI 2b (71%) and mTICI 0–2a (66%) (Table 1).

**TABLE 1:** Cohort baseline, procedural and outcome characteristics.

	mTICI 0–2a *N* = 650	mTICI 2b *N* = 1873	mTICI 3 *N* = 2925	Total *N* = 5448	*P*
Baseline characteristics					
Age	71.9 (13.0)	69.9 (13.6)	71.9 (12.8)	71.2 (13.1)	< 0.001
Sex (female)	330 (51%)	903 (48%)	1404 (48%)	2637 (48%)	0.433
NIHSS at admission	14 (8–18)	13 (8–17)	13 (8–18)	13 (8–18)	0.107
Hypertension	482 (74%)	1316 (70%)	2184 (75%)	3982 (73%)	0.003
Diabetes mellitus	129 (20%)	332 (18%)	604 (21%)	1065 (20%)	0.044
Dyslipidemia	230 (35%)	696 (37%)	1216 (42%)	2142 (39%)	< 0.001
Atrial fibrillation	217 (33%)	638 (34%)	1206 (41%)	2061 (38%)	< 0.001
Imaging					
ASPECTS at admission	8 (7–10)	9 (7–10)	9 (7–10)	9 (7–10)	< 0.001
Treatment characteristics					
Administration of tPA	319 (49%)	1033 (55%)	1566 (54%)	2918 (54%)	0.028
Anesthesia during treatment					
Local anesthesia	33 (5%)	72 (4%)	81 (3%)	186 (3%)	< 0.001
Sedation with local anesthesia	187 (29%)	470 (25%)	599 (20%)	1256 (23%)	< 0.001
General anesthesia	430 (66%)	1331 (71%)	2245 (77%)	4006 (74%)	< 0.001
Number of retrieval attempts	3.0 (2.5)	2.3 (1.6)	1.8 (1.2)	2.1 (1.6)	< 0.001
Final TICI‐Score					
0	329 (51%)	0 (0%)	0 (0%)	329 (6%)	< 0.001
1	79 (12%)	0 (0%)	0 (0%)	79 (1%)	< 0.001
2a	242 (37%)	0 (0%)	0 (0%)	242 (4%)	< 0.001
2b	0 (0%)	1873 (100%)	0 (0%)	1873 (34%)	< 0.001
3	0 (0%)	0 (0%)	2925 (100%)	2925 (54%)	< 0.001
SO/LSW to admission (min) Missings (*n*)	303.2 (376.9) 80	306.6 (420.3) 190	269.8 (326.0) 246	286.2 (367.0) 516	0.003
Admission to groin (min) Missings (*n*)	101.1 (124.1) 32	97.5 (136.2) 53	100.4 (158.8) 86	99.5 (147.5) 171	0.769
Clinical outcome					
mRS at 90 days (median, IQR)	5 (3–6)	3 (1–5)	2 (1–4)	3 (1–5)	< 0.001
Mean, SD	4.2 (1.9)	3.0 (2.1)	2.6 (2.1)	2.9 (2.1)	< 0.001
Good functional outcome (mRS 0–2)	131 (20%)	856 (46%)	1565 (54%)	2552 (47%)	< 0.001
Mortality (mRS = 6)	251 (39%)	383 (20%)	488 (17%)	1122 (21%)	< 0.001
Adverse events					
Perforation/Dissection	56 (9%)	51 (3%)	47 (2%)	154 (3%)	< 0.001
Embolic clot Migration	57 (9%)	117 (6%)	55 (2%)	229 (4%)	< 0.001
Intracranial Hemorrhage (ICH)	43 (7%)	82 (4%)	59 (2%)	184 (3%)	< 0.001
Symptomatic ICH (24 h)	34 (5%)	73 (4%)	51 (2%)	158 (3%)	< 0.001
Vasospasm	26 (4%)	99 (5%)	102 (3%)	227 (4%)	0.010

*Note:* Age, time metrics, number of passes, and mean mRS are presented as mean (SD); NIHSS, ASPECTS, and median mRS at 90 days are presented as median (IQR); categorical variables as *n* (%). Group comparisons were performed using the one‐way analysis of variance (ANOVA)test for continuous variables, Kruskal‐Wallis test for ordinal variables and the chi‐square test for categorical variables. Statistical significance was set at *p* < 0.05. Data were analyzed after propensity score matching.

Abbreviations: ASPECTS, Alberta Stroke Program Early CT Score; ICH, intracranial hemorrhage; IQR, interquartile range; LSW, last seen well; mRS, modified Rankin Scale; mTICI, modified Thrombolysis in Cerebral Infarction; NIHSS, National Institutes of Health Stroke Scale; SD, standard deviation; SO, symptom onset; tPA, tissue plasminogen activator.

### 3.2 Functional Outcome

Significant differences were observed for the primary endpoint of good functional outcome (mRS 0–2 at day 90) across recanalization grades (*p* < 0.001). Among patients with minimal or no reperfusion (mTICI 0–2a), only 20% achieved a good functional outcome, compared to 46% in the mTICI 2b group and 54% in those with complete recanalization (mTICI 3).

Median mRS scores at 90 days also differed significantly by recanalization status (*p* < 0.001), with the highest disability observed in the mTICI 0–2a group (median mRS 5, IQR 3–6), followed by mTICI 2b (median mRS 3, IQR 1–5), and the lowest in the mTICI 3 group (median mRS 2, IQR 1–4). Exploratory analysis of mean mRS confirmed these findings, with mean scores of 4.2 (SD 1.9), 3.0 (SD 2.1), and 2.6 (SD 2.1) for the mTICI 0–2a, 2b, and 3 groups, respectively (*p* < 0.001) (Table 1).

Using IPWRA estimators, the adjusted rates of good outcome (mRS 0–2) were 25% (95% CI: 22%–28%) for mTICI 0–2a, 46% (95% CI: 44%–48%) for mTICI 2b, and 52% (95% CI: 50%–54%) for mTICI 3. Consistent with these findings, the adjusted average mRS score at 90 days improved significantly with increasing recanalization success: 4.0 (95% CI: 3.8–4.1) for mTICI 0–2a, 3.0 (95% CI: 2.9–3.1) for mTICI 2b, and 2.7 (95% CI: 2.6–2.7) for mTICI 3 (Table 2).

**TABLE 2:** IPWRA outcome estimates for mean mRS and percentage of mRS 0–2 at day 90.

*Stratification*			% mRS 0–2 at 90 days (IPWRA)	mRS at 90 days (IPWRA)	% sICH (observed)	% Mortality mRS 6 at 90 days (IPWRA)
NIHSS	ASPECTS	mTICI	% (95%‐CI)	Mean (95%‐CI)	%	% (95%‐CI)
All	All	0–2a	25% (22%–28%)	3.97 (3.82–4.12)	5.8%	34% (30%–37%)
All	All	2b	46% (44%–48%)	2.98 (2.89–3.07)	4.2%	21% (19%–23%)
All	All	3	52% (50%–54%)	2.65 (2.58–2.73)	1.8%	17% (16%–19%)
0–10	10	0–2a	46% (35%–57%)	2.93 (2.48–3.38)	7.1%	20% (12%–27%)
0–10	10	2b	66% (60%–72%)	1.93 (1.71–2.15)	3.2%	9% (5%–12%)
0–10	10	3	74% (70%–78%)	1.67 (1.51–1.82)	1.4%	9% (7%–12%)
0–10	8–9	0–2a	38% (28%–48%)	3.01 (2.63–3.39)	4.1%	15% (9%–21%)
0–10	8–9	2b	70% (65%–75%)	2.02 (1.80–2.23)	4.5%	12% (9%–16%)
0–10	8–9	3	72% (67%–76%)	1.69 (1.50–1.87)	1.6%	7% (5%–10%)
0–10	≤ 7	0–2a	38% (28%–47%)	3.40 (2.94–3.85)	7.0%	20% (12%–27%)
0–10	≤ 7	2b	59% (51%–67%)	2.31 (2.03–2.59)	7.6%	9% (4%–14%)
0–10	≤ 7	3	63% (56%–71%)	2.04 (1.75–2.33)	1.6%	8% (3%–12%)
11–15	10	0–2a	28% (19%–37%)	4.13 (3.56–4.69)	3.7%	44% (31%–57%)
11–15	10	2b	54% (47%–62%)	2.69 (2.38–2.99)	1.4%	20% (14%–26%)
11–15	10	3	62% (57%–68%)	2.16 (1.96–2.37)	0.5%	10% (7%–13%)
11–15	8–9	0–2a	24% (14%–34%)	4.36 (3.94–4.77)	4.9%	34% (23%–45%)
11–15	8–9	2b	43% (37%–50%)	2.98 (2.72–3.23)	4.0%	16% (11%–21%)
11–15	8–9	3	54% (48%–59%)	2.49 (2.27–2.72)	1.7%	11% (7%–15%)
11–15	≤ 7	0–2a	19% (11%–27%)	4.22 (3.84–4.59)	8.2%	32% (24%–41%)
11–15	≤ 7	2b	31% (25%–38%)	3.57 (3.31–3.83)	4.2%	21% (14%–27%)
11–15	≤ 7	3	38% (31%–44%)	3.31 (3.04–3.57)	1.3%	22% (16%–28%)
≥ 16	10	0–2a	9% (3%–16%)	4.79 (4.42–5.16)	0.0%	53% (40%–65%)
≥ 16	10	2b	32% (25%–39%)	3.67 (3.37–3.98)	4.7%	35% (28%–42%)
≥ 16	10	3	47% (42%–53%)	2.92 (2.68–3.15)	2.3%	23% (19%–28%)
≥ 16	8–9	0–2a	11% (6%–17%)	4.65 (4.40–4.90)	5.6%	45% (37%–53%)
≥ 16	8–9	2b	29% (24%–35%)	3.70 (3.46–3.93)	3.6%	29% (23%–34%)
≥ 16	8–9	3	36% (31%–41%)	3.40 (3.19–3.62)	1.5%	26% (21%–30%)
≥ 16	≤ 7	0–2a	12% (6%–18%)	5.16 (4.89–5.44)	9.3%	56% (45%–66%)
≥ 16	≤ 7	2b	20% (16%–24%)	4.21 (4.01–4.41)	5.2%	39% (34%–45%)
≥ 16	≤ 7	3	23% (18%–27%)	4.06 (3.88–4.23)	3.7%	35% (30%–39%)

*Note:* This table presents the estimated mean probability of functional independence (mRS 0–2 at 90 days), as well as the estimated probabilities of symptomatic intracranial hemorrhage (sICH, observed) and mortality at 90 days, stratified by admission NIHSS, ASPECTS, and angiographic reperfusion success (final mTICI). Estimates for functional outcome and mortality are reported with 95% confidence intervals (CI) and were calculated using inverse‐probability‐weighted regression adjustment (IPWRA) to control for baseline confounders.

Abbreviations: ASPECTS, Alberta Stroke Program Early CT Score; mRS, modified Rankin Scale; mTICI, modified Thrombolysis in Cerebral Infarction Scale; NIHSS, National Institutes of Health Stroke Scale; SD, standard deviation; sICH, symptomatic intracranial hemorrhage.

### 3.3 Stratification by NIHSS and ASPECTS

**FIGURE 2:**
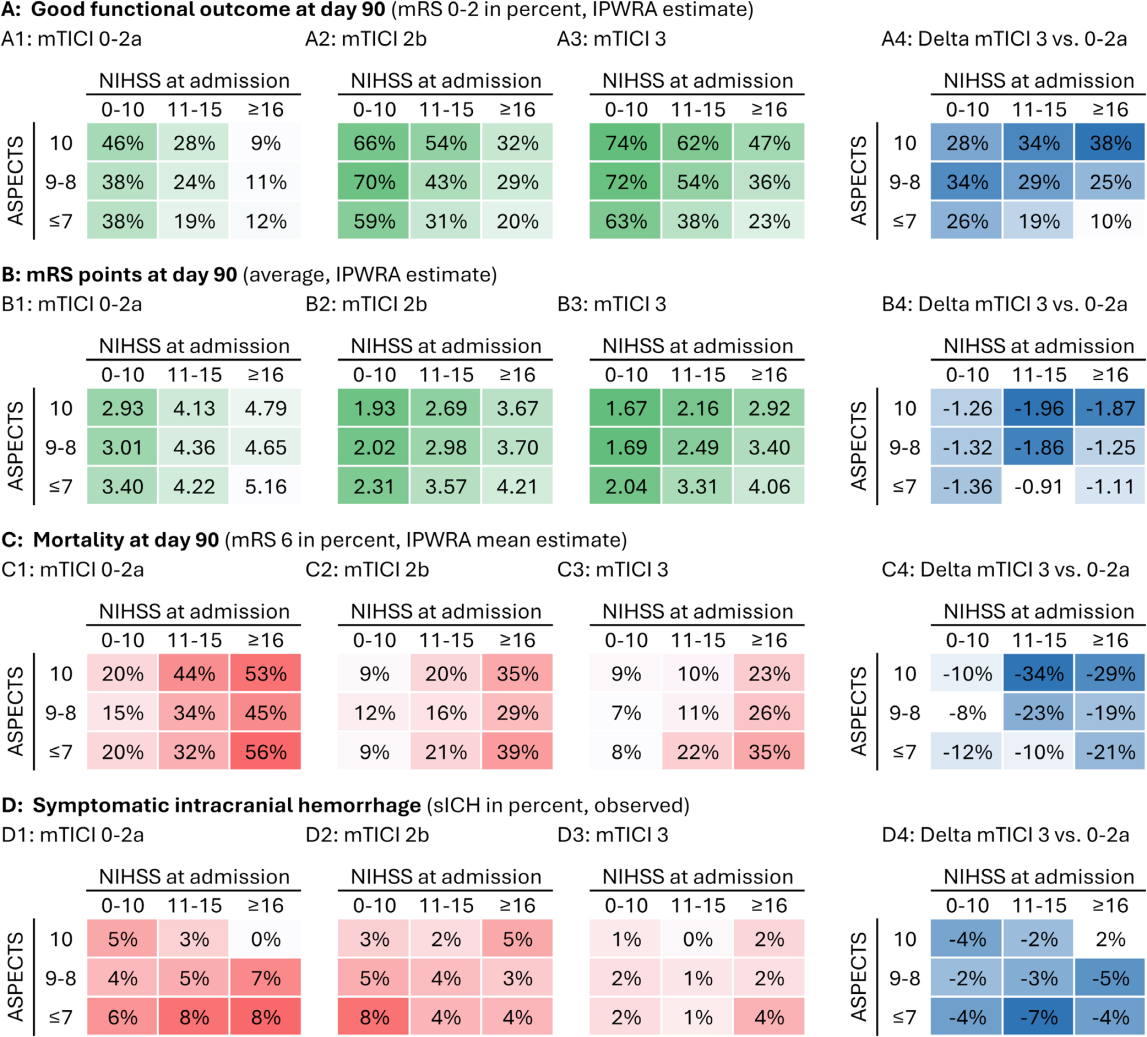
IPWRA‐estimated outcomes at day 90 stratified by NIHSS and ASPECTS at admission and by final mTICI grade. Shown are estimated probabilities of good functional outcome (mRS 0–2 at 90 days), average mRS scores, mortality, and symptomatic intracranial hemorrhage (sICH), stratified by baseline NIHSS, ASPECTS, and angiographic reperfusion success. Estimates for functional outcome and mortality include 95% confidence intervals (CI) and were calculated using inverse‐probability‐weighted regression adjustment (IPWRA) to control for baseline confounding. Color coding indicates outcome type: Green for functional outcome, red for adverse events (sICH, mortality), and blue for absolute differences in outcome between complete and unsuccessful reperfusion (mTICI 3 vs. 0–2a). Color intensity reflects effect magnitude. Abbreviations: MRS, modified Rankin Scale; NIHSS, National Institutes of Health Stroke Scale; ASPECTS, Alberta Stroke Program Early CT Score; mTICI, modified Thrombolysis in Cerebral Infarction; sICH, symptomatic intracranial hemorrhage; CI, confidence interval.

Stratified analysis showed that the benefit of achieving good functional outcome (mRS 0–2) after successful reperfusion (mTICI 3) was highest in patients with high stroke severity and high ASPECTS, reflecting a high clinical‐core mismatch; however, significant increases in rates of mRS 0–2 were observed across all subgroups. In the NIHSS ≥ 16 and ASPECTS 10 subgroup, 9% (95% CI: 3%–16%) of patients with mTICI 0–2a achieved mRS 0–2, compared to 47% (95% CI: 42%–53%) in the mTICI 3 group, which reflects an absolute difference of 38 percentage points. Comparable absolute differences in mRS 0–2 rates between mTICI 0–2a and mTICI 3 were observed in patients with NIHSS 11–15 and ASPECTS 10. However, significant differences in functional outcome were also found in patients with lower ASPECTS, indicating that the benefit of successful reperfusion extends to subgroups with larger established infarct cores. In the NIHSS ≥ 16 and ASPECTS ≤ 7 subgroup, the proportion of patients achieving mRS 0–2 was 12% (95% CI: 6%–18%) for mTICI 0–2a and 23% (95% CI: 18%–27%) for mTICI 3, which reflects an absolute difference of 11 percentage points (Figure 2) (Table 2). Similar findings emerged from the exploratory analysis of average mRS scores and perfusion‐related mRS shifts at 90 days, showing the greatest benefit of full recanalization (mTICI 3 vs. mTICI 0–2a) in the subgroup with ASPECTS 10 and NIHSS 11–15. In this group, the average 90‐day mRS was 2.92 after mTICI 3 recanalization compared to 4.79 following unsuccessful reperfusion, corresponding to a 1.96‐point reduction in mRS.

### 3.4 Comparison of mTICI 2b and mTICI 3 Outcomes

The highest increase in the probability of achieving good functional outcome (mRS 0–2) after mTICI 3 compared to mTICI 2b recanalization was observed in the NIHSS ≥ 16, ASPECTS 10 subgroup. In this subgroup, 32% (95% CI: 25%–39%) of patients with mTICI 2b achieved mRS 0–2 compared to 47% (95% CI: 42%–53%) with mTICI 3, accounting for an absolute difference of 15 percentage points. In patients with large signs of infarction (NIHSS ≥ 16, ASPECTS ≤ 7), the estimated probability of achieving mRS 0–2 was 20% (95% CI: 16%–24%) for mTICI 2b and 20% (95% CI: 16%–24%) for mTICI 3, indicating no significant beneficial effect of mTICI 3 compared to mTICI 2b. Similarly small differences in mRS 0–2 rates between mTICI 3 and 2b were observed in the NIHSS 0–10 subgroup across ASPECTS 8–9 and ASPECTS 10 (Figure 2) (Table 2).

### 3.5 Safety Outcomes

sICH occurred in 34/650 (5%) patients with mTICI 0–2a, in 73/1873 (4%) patients with mTICI 2b and in 51/2925 (2%) patients with mTICI 3; group comparison confirmed significant differences (*p* < 0.001). Mortality rates at 90 days were 17% in the mTICI 3 group, 20% in the mTICI 2b group and 39% in the mTICI 0–2a group, *p* < 0.001 (Table 1). Based on IPWRA estimators, the most pronounced mortality reduction following mTICI 3 recanalization vs. mTICI 0–2a of a 34 percentage points risk reduction was observed in patients with NIHSS 11–15 and ASPECTS 10, while the lowest beneficial effect was estimated for patients with low NIHSS and low ASPECTS, where full recanalization led to a 10 percentage points reduction in mortality (Figure 2) (Table 2).

Other procedure‐related adverse events included vasospasm (4%), arterial dissection or perforation (3%), clot migration and embolization (4%), and intracerebral hemorrhage (3%). Their occurrence was inversely correlated with the degree of recanalization success. Dissections or perforations were most frequent in the mTICI 0–2a group (9%), compared to 3% in mTICI 2b and only 2% in mTICI 3 (*p* < 0.001) (Table 1).

## 4 Discussion

This study based on large‐scale multicenter registry data evaluated the effect of successful reperfusion on functional outcome following mechanical thrombectomy in acute ischemic stroke patients, stratified by NIHSS and ASPECTS scores at admission, reflecting clinical‐core mismatch. In a cohort of 5448 patients, successful reperfusion was significantly associated with improved functional outcomes across all subgroups. However, the effect of reperfusion on outcome varied according to baseline NIHSS and ASPECTS strata. The greatest benefit of successful reperfusion was observed in patients with a high clinical‐core mismatch, particularly those with ASPECTS 10 and NIHSS scores > 15. Importantly, our findings do not support exclusion of patients from thrombectomy based on NIHSS‐ASPECTS ratios, but rather indicate that mismatch profiles primarily modify effect size instead of defining treatment eligibility. This is consistent with results observed in previous randomized trials, including DEFUSE‐3 [17] and DAWN [10], which reported improved clinical outcomes in patients exhibiting a pronounced clinical‐core mismatch, even when treated within extended time windows of 6 to 16 h (DEFUSE‐3) and 6 to 24 h (DAWN). In the DAWN trial, mismatch was defined as a severe clinical deficit (NIHSS ≥ 10 or ≥ 20) combined with a small infarct core of 21–51 mL, depending on age (≥ 80 vs. < 80 years) and NIHSS (≥ 10 or ≥ 20), as determined by CT perfusion or DWI [10]. In the DEFUSE‐3 trial, mismatch was defined by perfusion imaging, requiring a small core (< 70 mL), a mismatch ratio ≥ 1.8, and an absolute mismatch of at least 15 mL [17]. Unlike these trials, our study did not utilize volumetric analysis, but employed NIHSS and ASPECTS at admission as a surrogate for the assessment of the clinical‐core mismatch. Use of this surrogate definition may introduce misclassification compared with perfusion‐based or volume‐based approaches and may thereby reduce apparent differences between mismatch subgroups.

While patients with high clinical‐core mismatch benefited the most, estimated average effects of recanalization were lower in cases with minor deficits or extensive signs of infarct. Thus, patients with ASPECTS scores of 8–9 and ≤ 7 also showed significant, though progressively smaller effects. This aligns with recent RCT findings demonstrating that even patients with large infarcts benefit from endovascular therapy [20]. Consistent with these findings, the SELECT2 trial showed that patients with large infarcts, defined as ASPECTS 3–5 or core volumes > 50 mL, benefit from thrombectomy even in the absence of a pronounced clinical‐core mismatch [4].

Also, patients with milder symptoms (NIHSS 0–10) showed positive but less pronounced functional outcome gains. Further subdivision into NIHSS 0–5 and 6–10 could provide additional granularity but was not feasible in the present analysis and should be addressed in future studies. Their smaller initial deficits suggest a reduced penumbral volume, which in turn diminishes the relative impact of recanalization compared to patients with more severe deficits. Notably, even patients with low clinical‐core mismatch showed measurable, clinically relevant improvements after recanalization in all stratified subgroups. In contrast to these findings, the ANGEL‐ASPECT trial did not demonstrate a significant benefit of endovascular therapy in patients with a clinical‐radiological mismatch, defined in the trial as high NIHSS (≥ 16) with a small infarct core (< 70 mL) or low NIHSS (< 16) with a large infarct core (≥ 70 mL) [21]. The authors suggest that this may be due to strategic infarct locations with little salvageable tissue in the high NIHSS/small core group, and non‐eloquent infarct regions in the low NIHSS/large core group, limiting the potential for functional improvement despite reperfusion [21]. These differences suggest that concepts of clinical‐core or perfusion mismatch do not fully explain treatment response [22]. Recent randomized trials in distal medium vessel occlusions (DISTAL, ESCAPE‐MeVO, DISCOUNT) further emphasize that mismatch‐based paradigms may not uniformly translate across vascular territories [23, 24, 25].

Outcome improvements after full recanalization (mTICI 3) compared to near‐complete recanalization (mTICI 2b) varied across subgroups. Patients with high clinical‐core mismatch (ASPECTS 10 and NIHSS ≥ 16) benefited the most from mTICI 3 compared to mTICI 2b, with a 0.75‐point reduction in mRS at 90 days, as observed in other studies [16, 26, 27, 28]. Our findings underline the benefit of achieving complete reperfusion (mTICI 3), especially in patients with a high clinical‐core mismatch, as reported in previous analyses [26, 27]. However, in subgroups with low mismatch, such as those with high ASPECTS (10) and low NIHSS (0–10), or low ASPECTS (**≤** 7) and high NIHSS (**≥** 16), the additional functional benefit of mTICI 3 over near‐complete reperfusion (mTICI 2b) appears less pronounced, consistent with recent findings [29]. Considering the increased risk of periprocedural complications associated with multiple retrieval attempts, a pragmatic approach that accepts mTICI 2b as an adequate procedural endpoint may be appropriate in selected cases [16, 29].

In summary, our findings emphasize the need for an individualized approach to thrombectomy decision‐making, especially if resources are limited. While all analyzed subgroups showed a significant benefit from successful reperfusion, the effect size varied across subgroups, with the most favorable outcome profiles observed in patients with high clinical‐core mismatch. In patients with borderline eligibility, such as those presenting with high NIHSS scores but low ASPECTS, population‐level data indicate a substantial benefit from successful reperfusion. Findings in high clinical‐core mismatch profiles confirm prior randomized trial evidence, whereas observations in mild stroke and low‐ASPECTS groups should be considered exploratory. However, further research is needed to refine patient selection criteria and distinguish those who are likely to benefit from mechanical thrombectomy from those for whom best medical management may be more appropriate. Such refinement will require approaches that move beyond fixed subgroup thresholds toward continuous, individualized risk estimation. Furthermore, the development of individualized prediction models was beyond the scope of this study but represents an important future direction.

Future studies should focus on optimizing selection criteria by integrating advanced imaging biomarkers, such as net water uptake (NWU) [30, 31], to more accurately identify patients likely to derive benefit from successful reperfusion, particularly in resource‐limited settings where access to endovascular therapy is constrained.

Our study has limitations. The conducted complete case analysis may have introduced bias by excluding patients with missing data and could reduce the generalizability of our findings. Approximately 70% of screened patients were excluded due to missing key variables, which represents a substantial selection process and may have influenced effect estimates and limit direct generalizability to unselected thrombectomy populations. Exclusion of patients with pre‐stroke disability may further limit generalizability. Estimates of outcome differences across recanalization grades are based on large‐scale registry data, which may be affected by confounding variables. IPWRA estimators were used to control for confounders in observational data. However, unmeasured factors, such as collateral circulation, infarct location and infarct progression, may introduce bias to the analysis. In addition, variability in onset to imaging time, which is known to influence ASPECTS sensitivity and outcome, could not be accounted for and represents a potential source of residual confounding error. The use of linearized mRS scores in the exploratory analysis assumes equal spacing between scale points, which may not fully capture the ordinal nature and clinical relevance of functional changes. As such, results should be interpreted in the context of the presented categorical analyses. Finally, assessment of ASPECTS on admission, mTICI grading and the documentation of retrieval attempts were performed locally by the treating interventionalist and/or radiologist, which may have introduced additional observer‐related bias.

## 5 Conclusion

This study based on multicenter registry data including 5448 patients confirms significant beneficial effects of successful reperfusion following mechanical thrombectomy across different clinical profiles stratified by ASPECTS and NIHSS, including both high and low clinical‐core mismatch groups. However, outcome patterns differed across subgroups, with the greatest benefit observed in patients with a high clinical‐core mismatch. Although subgroup cutoffs were chosen for clinical interpretability, outcome effects across ASPECTS likely follow a continuous rather than stepwise pattern. For patients with borderline eligibility, such as those presenting with low NIHSS, the decision to pursue full recanalization should be carefully balanced against the potential risk of treatment‐related complications.

## Author Contributions

F.S. and H.C.K. conceptualized and designed the study and interpreted the data. H.C.K. performed the statistical analyses and prepared the figures. F.S. drafted the manuscript. L.M., G.B., M.B., C.T., C.H., L.W., V.G., A.H., M.J., P.G., L.M., T.F., J.N., C.B., F.F., M.S., U.H., G.T., J.F., and S.G. acquired the data, contributed to data validation, including assessment of clinical and imaging parameters used in the final analysis. All authors critically revised the manuscript for important intellectual content, approved the final version to be published, and agreed to be accountable for all aspects of the work.

## Funding

The authors have nothing to report.

## Conflicts of Interest

Helge Kniep reports compensation as speaker from Asklepios Kliniken, travel funding from Penumbra, an ownership stake in Eppdata GmbH, and compensation from Eppdata GmbH for consultant services. Fabian Flottmann is a consultant for Eppdata GmbH. Tobias Faizy has received research grants from the Deutsche Forschungsgemeinschaft/German Research Foundation. Götz Thomalla received fees as consultant from Acandis, Boehringer Ingelheim, Bayer, and Portola, and fees as lecturer from Acandis, Alexion, Amarin, Bayer, Boehringer‐Ingelheim, BMS/Pfizer, Daiichii Sankyo, and Portola. He serves on the board of the TEA Stroke Study and of ESO. Jens Fiehler is a consultant for Cerenovus, Medtronic, Microvention, Penumbra, Phenox, Roche, and Tonbridge. He serves on the advisory board of Stryker and Phenox. He is a stockholder of Tegus Medical, Eppdata, and Vastrax. He serves as Associate Editor at JNIS.

## German Stroke Registry—Endovascular Treatment (GSR‐ET)—Steering Committee

Alexander Nave; Anna Alegiani; Annerose Mengel; Arno Reich; Bernd Eckert; Charlotte Pietrock; Christian Nolte; Eberhard Siebert; Fabian Flottmann; Fee Keil; Franziska Dorn; Gabor Petzold; Götz Thomalla; Hanna Zimmermann; Jan Borggrefe; Jan Hendrik Schäfer; Jens Fiehler; Joachim Röther; Klaus Gröschel; Lars Kellert; Mario Abruscato; Maximilian Schell; Omid Nikoubashman; Peter Schellinger; Silke Wunderlich; Steffen Tiedt; Sven Thonke; Timo Uphaus; Tobias Boeckh‐Behrens; Ulrike Ernemann.

## Data Availability

The data that support the findings of this study are available upon reasonable request after approval of the German Stroke Registry Endovascular Treatment (GSR‐ET).
